# Percent amplitude of fluctuation: A simple measure for resting-state fMRI signal at single voxel level

**DOI:** 10.1371/journal.pone.0227021

**Published:** 2020-01-08

**Authors:** Xi-Ze Jia, Jia-Wei Sun, Gong-Jun Ji, Wei Liao, Ya-Ting Lv, Jue Wang, Ze Wang, Han Zhang, Dong-Qiang Liu, Yu-Feng Zang

**Affiliations:** 1 Center for Cognition and Brain Disorders, Institutes of Psychological Sciences, Hangzhou Normal University, Hangzhou, Zhejiang, China; 2 Zhejiang Key Laboratory for Research in Assessment of Cognitive Impairments, Hangzhou, Zhejiang, China; 3 School of Information and Electronics Technology, Jiamusi University, Jiamusi, Heilongjiang, China; 4 Department of Medical Psychology, Chaohu Clinical Medical College, Anhui Medical University, Hefei, China; Center for Neuroscience and Regenerative Medicine, UNITED STATES

## Abstract

The amplitude of low-frequency fluctuation (ALFF) measures resting-state functional magnetic resonance imaging (RS-fMRI) signal of each voxel. However, the unit of blood oxygenation level-dependent (BOLD) signal is arbitrary and hence ALFF is sensitive to the scale of raw signal. A well-accepted standardization procedure is to divide each voxel’s ALFF by the global mean ALFF, named mALFF. Although fractional ALFF (fALFF), a ratio of the ALFF to the total amplitude within the full frequency band, offers possible solution of the standardization, it actually mixes with the fluctuation power within the full frequency band and thus cannot reveal the true amplitude characteristics of a given frequency band. The current study borrowed the percent signal change in task fMRI studies and proposed percent amplitude of fluctuation (PerAF) for RS-fMRI. We firstly applied PerAF and mPerAF (i.e., divided by global mean PerAF) to eyes open (EO) vs. eyes closed (EC) RS-fMRI data. PerAF and mPerAF yielded prominently difference between EO and EC, being well consistent with previous studies. We secondly performed test-retest reliability analysis and found that (PerAF ≈ mPerAF ≈ mALFF) > (fALFF ≈ mfALFF). Head motion regression (Friston-24) increased the reliability of PerAF, but decreased all other metrics (e.g. mPerAF, mALFF, fALFF, and mfALFF). The above results suggest that mPerAF is a valid, more reliable, more straightforward, and hence a promising metric for voxel-level RS-fMRI studies. Future study could use both PerAF and mPerAF metrics. For prompting future application of PerAF, we implemented PerAF in a new version of REST package named RESTplus.

## 1. Introduction

The neuroimaging field has an increasing research interest in assessing the spontaneous brain activity using BOLD RS-fMRI. Since the first RS-fMRI study [1], functional connectivity (FC) is still the most widely used method in the RS-fMRI studies. However, by focusing on temporal synchronization of the BOLD signals between any pair of brain regions, FC analysis cannot directly measure the regional spontaneous brain activity of a specific voxel per se. Another widely used RS-fMRI measurement, ALFF [2], can be adopted on this purpose, as it provides direct characterization of the spontaneous brain activity at each voxel. However, since BOLD signal has arbitrary units, ALFF is sensitive to the scale of the raw BOLD signal and cannot be directly applied to subsequent group-level statistical analysis.

An approach to deal with such a scaling effect is to normalize the raw amplitude value by the global mean amplitude, i.e., the averaged amplitude value across all voxels in the brain [2–4]. However, such manipulation assumes that the BOLD signal of all voxels has the same scaling value. In fact, many factors affect the scaling value, including spatial inhomogeneity, brain tissue composition, and brain lesions. Standardization at single voxel level may be helpful to reduce such effects.

Another voxel-level metric, namely fractional ALFF (fALFF), had been proposed to normalize ALFF[5]. The fALFF is a ratio of the ALFF within a specific low frequency band (usually 0.01–0.08 Hz) to the total BOLD fluctuation amplitude within the full frequency band [5]. It can be regarded as a standardized ALFF-like metric at the single voxel level and is theoretically a scale-independent (i.e., not depending on the mean absolute value of the BOLD signals) method. One-sample t-tests on fALFF within a group of participants did confirm that a much better default mode network pattern could be captured by fALFF (i.e., significantly higher fALFF within the default mode network) compared to ALFF [5]. Previous study have also shown that the RS-fMRI can significantly reduce inter-subject variability of task fMRI activations [6]. However, changes in any frequency band will result in the fALFF change of the frequency of interest. Moreover, previous studies have shown that fALFF had generally lower test-retest reliability than that of ALFF in gray matter voxels [4,7]. Recently, frequency-dependent or frequency-specific analysis of RS-fMRI has been drawing increasing attention [8–14]; many studies have suggested that frequency-specific BOLD fluctuations can be used to detect disease biomarkers [15,16] and to detect different subject’s status [17]. From the above analysis, fALFF may not be a proper standardized metric for frequency-specific studies.

In task fMRI studies, percent signal change is a popular metric of task-induced BOLD signal changes, i.e., the difference in fMRI signal between the baseline condition (B) and the task condition (T), here, percent signal change = (T-B)/B×100%. Typical percent signal change is approximately 1% - 3% in block design [18,19] and from 0.2% to 1% in event-related design[20,21]. Although RS-fMRI data has no such explicit task vs. control design, a similar metric to the percent signal change can be formulated for RS-fMRI by measuring the percentage of BOLD fluctuations relative to the mean BOLD signal intensity for each time point and averaging across the whole time series, namely “Percentage Amplitude Fluctuation” or PerAF. As compared with ALFF, PerAF is a scale-independent method. Both PerAF and mPerAF could be used for group-level statistical analysis. PerAF (mPerAF) can also avoid from the confounding mixture from voxel-specific fluctuation amplitude in fALFF. Hence, PerAF (mPerAF) seems to be a promising metric of voxel-level spontaneous BOLD activity.

The current study included 4 parts. 1) We first provided detailed formation of PerAF by using simulated data. 2) We applied PerAF (mPerAF) to detect voxel-level differences between two resting states (eyes open [EO] vs. eyes closed [EC]) because the differences between EO and EC have been consistently reported in previous RS-fMRI studies [17,22–26]. 3) We further assessed the test-retest reliability of PerAF (mPerAF). 4) To facilitate future applications, we implemented PerAF in a new version of REST package [27] named RESTplus [28] which based on Statistical Parametric Mapping (SPM) [29] and DPARSF [30]. We also made a command-line for calculating PerAF in another popular toolbox named Analysis of Functional Neuro-Images (AFNI) [31].

## 2. Methods and results

### 2.1. PerAF calculation and experiment on simulated data

The PerAF of each voxel was calculated as follows,

$$PerAF=\frac{1}{n}\sum_{i=1}^{n}|\frac{X_{i}-\mu }{\mu }|\times 100\%$$ (1)

$$\mu =\frac{1}{n}\sum_{i=1}^{n}X_{i}$$ (2)

where *X*_*i*_ is the signal intensity of the *i*_*th*_ time point, *n* is the total number of time points of the time series, and *μ* is the mean value of the time series.

**Fig 1:**
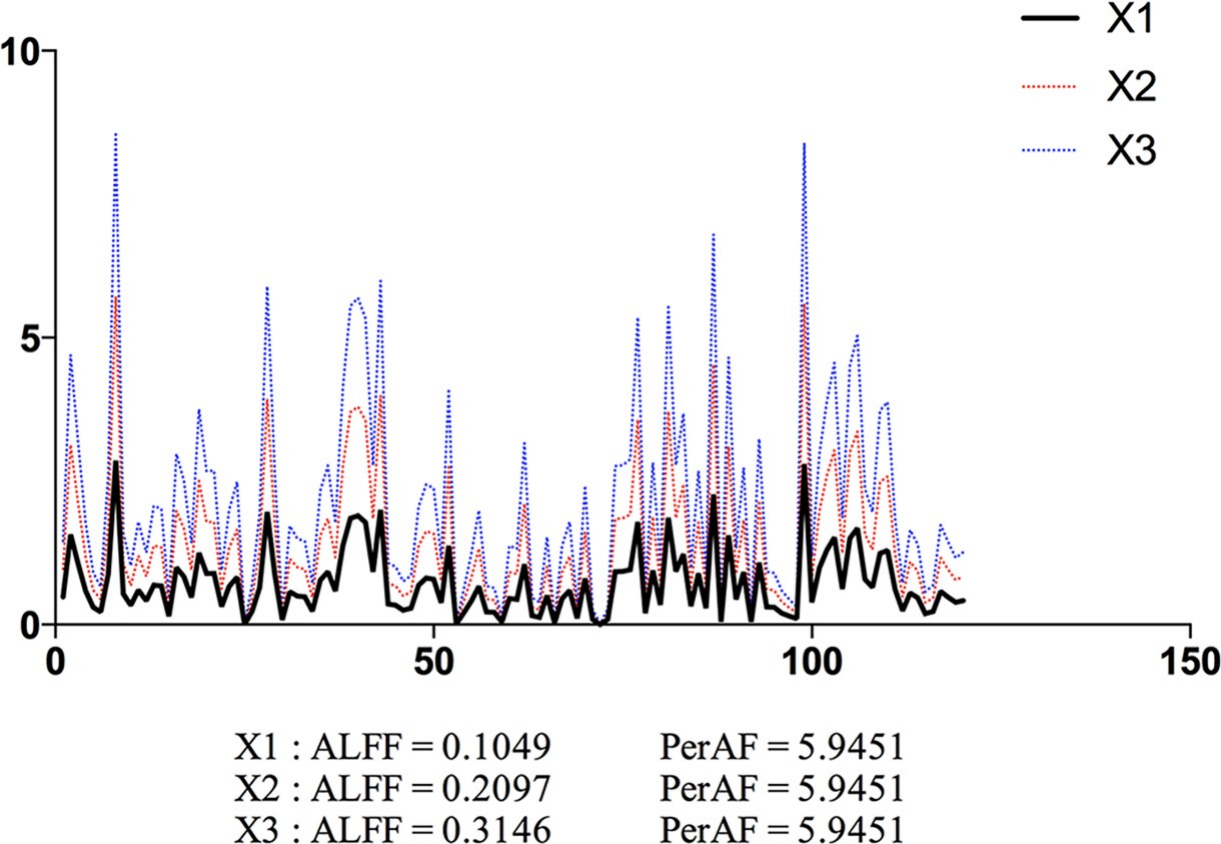
Simulated “resting-state” BOLD time series. The ALFF and PerAF of a simulated time series (X1), and its derivative time series of multiplying A by 2 (X2) and by 3 (X3). ALFF and PerAF were calculated for each time series. ALFF value is proportional to the mean value of the time series, but PerAF keeps the same. X and y axis are time and intensity, respectively, in arbitrary unit.

A simulated time series *X*_*1*_ was created, which contained 120 time points with random values. Its derivative time series *X*_*2*_ and *X*_*3*_ were generated by multiplying *X*_*1*_ by *2* and by 3, respectively (Fig 1). PerAF were calculated for each time series. For comparison purpose, we also calculated ALFF (see Section “2.2.3” for detailed ALFF calculation). As shown in Fig 1, the ALFF value is proportional to the mean value of the time series, but PerAF is not. Because the absolute BOLD signal intensity has arbitrary units, ALFF results will be affected by the scale of BOLD signal. The simulated data showed that, without a standardization procedure, ALFF can not be used for direct comparison.

### 2.2. Dataset-1: Comparison between EO and EC

#### 2.2.1. Data description

Dataset-1 was from a published dataset [22] which included 34 healthy participants (aged 19–31 years, 18 females). That study was approved by the Ethics Committee of the Center for Cognition and Brain Disorders, Hangzhou Normal University. Informed consent was obtained from each participant.

For each participant, four resting state sessions were scanned with two conditions EO and EC by BOLD and arterial spinlabeling (ASL), respectively. The order of the four sessions was counterbalanced across participants. The ASL data were not used in the current study. Dataset-1 was acquired using a GE MR-750 3.0 T scanner (GE Medical Systems, Waukesha, WI) at the Center for Cognition and Brain Disorders of Hangzhou Normal University. Each scan consisted of 240 contiguous EPI functional volumes (TR = 2000 ms; TE = 30 ms; flip angle = 60°; 37 axial slices; field of view (FOV) = 220 × 220 mm^2^, matrix = 64 × 64; in-plane resolution 3.44 × 3.44 × 3.4 mm^3^. For spatial normalization, a spoiled gradient-recalled pulse sequence was also used (176 sagittal slices; slice thickness = 1 mm; TR = 8100 ms; TE = 3.1 ms; flip angle = 9°; FOV = 250 × 250 mm^2^).

#### 2.2.2. Data preprocessing

The preprocessing was performed using Data Processing Assistant for Resting-State fMRI (DPARSF V2.3) [30] (http://www.restfmri.net), including: 1) discarding the first 10 timepoints for the longitudinal magnetization to reach a steady state and for participant’s adaptation to the scanning noise; 2) slice timing correction; 3) head motion correction; 4) co-registration, spatial normalization and resampling to 3 mm isotropic voxel size; 5) spatial smoothing with an isotropic Gaussian kernel with a FWHM of 6 mm; 6) removing the linear trend of the time series; 7) to remove the nuisance signals, the Friston 24-parameter model was used for covariate regression [32,33]; and 8) band-pass (0.01–0.08Hz) filtering for PerAF (but not for ALFF and fALFF). No participant was excluded from further analyses because of excessive head motion (more than 2.0 mm of maximal translation or 2.0° of maximal rotation).

#### 2.2.3. Paired t-test between EO and EC for PerAF, ALFF, and fALFF

The PerAF was calculated in the way as mentioned in section “2.1”. The ALFF and fALFF analysis was performed using REST [27] (http://www.restfmri.net). After preprocessing, the time series for each voxel was transformed into the frequency domain with a fast Fourier transform (FFT) and the power spectrum was then obtained. Since the power of a given frequency is proportional to the square of the amplitude of this frequency component, the square root was calculated at each frequency of the power spectrum and the averaged square root was obtained across 0.01–0.08 Hz at each voxel. This averaged square root was taken as the ALFF [2]. Then a ratio of the sum of amplitude within the low frequency band (i.e., ALFF) to that of the whole frequency band was computed as fALFF [5].

For standardization purpose, the ALFF of each voxel was divided by the global mean ALFF of each participant [2] (named mALFF hereafter). The same procedure was performed for fALFF [5] (named mfALFF hereafter). The PerAF of each voxel was also divided by the global mean PerAF of each participant, thus we have both PerAF and mPerAF. It has been proposed that z-transformation of ALFF could improve the normality of distribution acorss subjects [4]. Therefore we also transformed the PerAF, ALFF, and fALFF to their respective z score maps, i.e., minus by global mean and divided by standard deviation (SD), thus generating zPerAF, zALFF, and zfALFF. The different metrics and their derivatives were summarized in Table 1. As the original ALFF value is not suitable for comparison, it was excluded from further analysis. In case not every participant’s whole brain was completely covered, we made an intersection mask within which all 68 scanning sessions (2 sessions for each of the 34 participants) were covered (S1 Fig). Specifically, the mean fMRI image of each session was spatially normalized and then binarized (using logical function from MATLAB). Then all binary images and a whole brain mask which provided in the software REST [27] were combine to the intersection mask. The following statistical analysis was constrained within this intersection mask.

**Table 1:** Standardization methods in the current study.

Analytic method	
PerAF	without normalizing the signal by the global mean
mPerAF	divided by the global mean PerAF
zPerAF	minus global mean, then divided by SD
mALFF	divided by the global mean ALFF
zALFF	minus global mean, then divided by SD
fALFF	without normalizing the signal by the global mean
mfALFF	divided by the global mean fALFF
zfALFF	minus global mean, then divided by SD

Considering the head motion may affect the PerAF difference between EO and EC. To test this assumption, we calculated the head motion amount. To compare the amount of head motion between EO and EC, we calculated framewise displacement head motion [34]. Framewise head motion calculates the relative head motion of each timepoint to its prior timepoint. Zang and colleagues used the sum of framewise head motion of rotation and transition separately [2] (See formuli 1 and 2 in that reference). Power and colleagues integrated the sum of 6 framewise headmotion parameters as a whole, named framewise displacement (FD) [34]. Paired t-test was performed on FD between EO and EC. The FD was 0.1036 ± 0.0331 (mean ± standard deviation) for EO, and 0.1095 ± 0.0514 for EC. There was no significant difference (P = 0.3068).

Paired t-tests were performed between EO and EC within the intersection mask. The Gaussian Random Field (GRF) theory was used for multiple comparison correction. A combination of individual voxel’s P value < 0.05 and cluster P < 0.05 was used. The GRF analysis was performed using DPABI [35]. In addition, to view potential differences between EO and EC outside the brain, the results of paired t-test for PerAF map (i.e., without standardization by global mean PerAF) was also shown without multiple comparison correction, i.e., only a voxel-level P < 0.05 without cluster size threshold was adopted.

**Fig 2:**
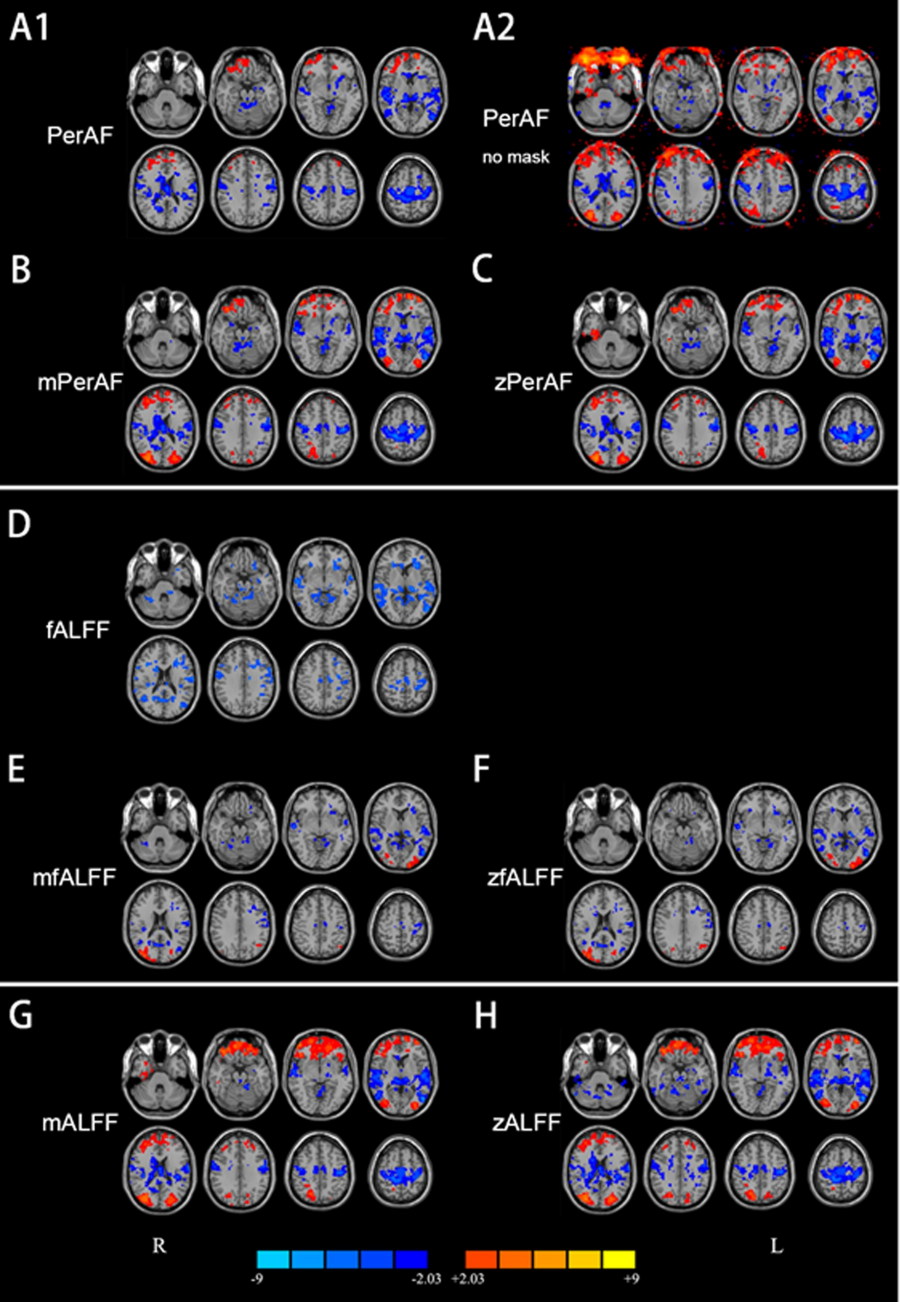
Results of paired t-test between eyes open (EO) and eyes closed (EC). A1: PerAF (without standardization by global mean) within brain intersection mask (P<0.05, corrected). A2: PerAF (without standardization by global mean) without brain mask (i.e., in the entire bounding box) (P<0.05, uncorrected). B: mPerAF (divided by global mean value) (P<0.05, corrected). C: zPerAF (minus mean then divided by standard deviation) (P<0.05, corrected). D—F: fALFF, mfALFF, and zfALFF, respectively (P<0.05, corrected). G, H: mALFF and zALFF, respectively (P<0.05, corrected). Warm colors indicate higher fluctuation in EO than EC, and cold colors indicate the opposite. L: left side of the brain. R: right side of the brain.

In the case without standardization by global mean, significantly lower (corrected for multiple comparisons) PerAF in EO than in EC was observed in widely distributed brain regions including the bilateral primary sensorimotor cortex (PSMC), supplementary motor area (SMA), paracentral lobule, primary auditory cortex extending to superior and middle temporal gyrus, thalamus, precuneus, visual cortex, and posterior cingulate cortex (P < 0.05, corrected) (Fig 2A1). Only small part of brain area (e.g., inferior orbital frontal, gyrus rectus) showed significantly higher PerAF in EO than EC.

For fALFF (In the case without standardization by global mean), the pattern of difference between EO and EC was similar with that of PerAF, but with smaller volume for most clusters (Fig 2A1 and 2D).

In the cases with global mean standardization, the between-condition difference of mPerAF (Fig 2B), zPerAF (Fig 2C), mALFF (Fig 2G), zALFF (Fig 2H) were very similar. Significantly higher fluctuation in EO than in EC was found in the bilateral middle occipital gyrus and orbitofrontal cortex. Significantly lower fluctuation in EO than in EC was found in the bilateral PSMC, SMA, paracentral lobule, thalamus, and primary auditory cortex (P < 0.05, corrected). All these results were highly consistent with previous studies [17,22–26]. For mfALFF (Fig 2E) and zfALFF (Fig 2F), the pattern of difference between EO and EC was generally similar with that of mPerAF, zPerAF, mALFF, and zALFF, except for that in the frontal pole and PSMC. mfALFF and zfALFF showed almost no difference in the frontal pole, while mPerAF, zPerAF, mALFF, and zALFF showed a big cluster. The cluster in the PSMC detected by mfALFF and zfALFF was smaller than that by mPerAF, zPerAF, mALFF, and zALFF.

The results of EO versus EC showed prominent inconsistency for comparisons with and without global mean standardization for PerAF (vs. mPerAF) as well for fALFF (vs. mfALFF) (Fig 2A1 vs. 2B, Fig 2D vs. 2E). Specifically, in the case of no global mean standardization, only a small area showed higher fluctuation in EO than in EC. However, after standardization, a few other areas showed significantly higher fluctuation in EO than in EC, including the bilateral middle occipital gyrus and a large area in the orbitofrontal cortex. (Not applicable for mfALFF and zfALFF results). Brain areas showing significantly lower fluctuation in EO than EC were slightly smaller than those without standardization. The prominent inconsistency suggested that the global mean PerAF had strong effect. We therefore performed a paired t-test on the global mean PerAF between the EO and EC. The global mean PerAF was calculated within a brain mask provided in REST [27]. There is no significant difference of the global mean PerAF between EO and EC (P = 0.8).

When no brain mask was used and no multiple comparison correction was performed, the eyeballs showed significantly higher PerAF in EO than EC (Fig 2A2). The difference in eyeballs extended to a large area of the frontal scalp and even to the orbitofrontal cortex.

### 2.3. Dataset-2: Test-retest reliability

#### 2.3.1. Data description

Dataset-2 is an open dataset from the International Neuroimaging Data-Sharing Intiative (INDI) (http://www.nitrc.org/projects/nyu_trt). This dataset includes 25 participants (mean age 30.7 ± 8.8 years, 16 females). The study was approved by the Institutional Review Board of the New York University School of Medicine and New York University. Also the study was approved by the Ethics Committee of the Center for Cognition and Brain Disorders, Hangzhou Normal University. Informed consent was obtained from each participant. Participants had three resting state sessions. Session 2 and 3 were collected 45 min apart, and were 5–16 months (mean 11 ± 4 months) after Session 1. During each scanning session, participants were instructed to continuously keep eyes open and a word “Relax” was centrally projected in white against a black background.

Dataset-2 was obtained using a 3T Siemens (Allegra) scanner. Each scan consisted of 197 contiguous EPI functional volumes (TR = 2000 ms; TE = 25 ms; flip angle = 90°; 39 axial slices; field of view (FOV) = 192 × 192 mm^2^; matrix = 64 × 64; acquisition voxel size = 3 × 3 × 3 mm^3^). For more information regarding Dataset-2 collection, please refer to [36].

#### 2.3.2. Data preprocessing

The data preprocessing steps were the same as mentioned in section “2.2.2”. Three participants were excluded from further analyses because of excessive head motion (more than 2.0 mm of maximal translation or 2.0° of maximal rotation) throughout the course of scanning.

In the original paper reporting Dataset-2, the authors did not use the cerebellum and inferior part of temporal lobe because these brain areas in some participants were not covered [36]. Therefore, we made an intersection mask within which all 75 scanning sessions (3 sessions for each of the 25 participants) were covered (S2 Fig). The detailed method was the same as in “2.2.3”. To compare the amount of head motion among different sessions, we calculated framewise displacement head motion [34]. One-way ANOVA was performed on FD among three sessions. The FD was 0.1530 ± 0.0727 (mean ± standard deviation) for Session 1, 0.1574 ± 0.0749 for Session 2, and 0.1704 ± 0.0740 for Session 3. There was no significant difference (P = 0.3313).

#### 2.3.3. Test-retest reliability of PerAF, ALFF, and fALFF

**Fig 3:**
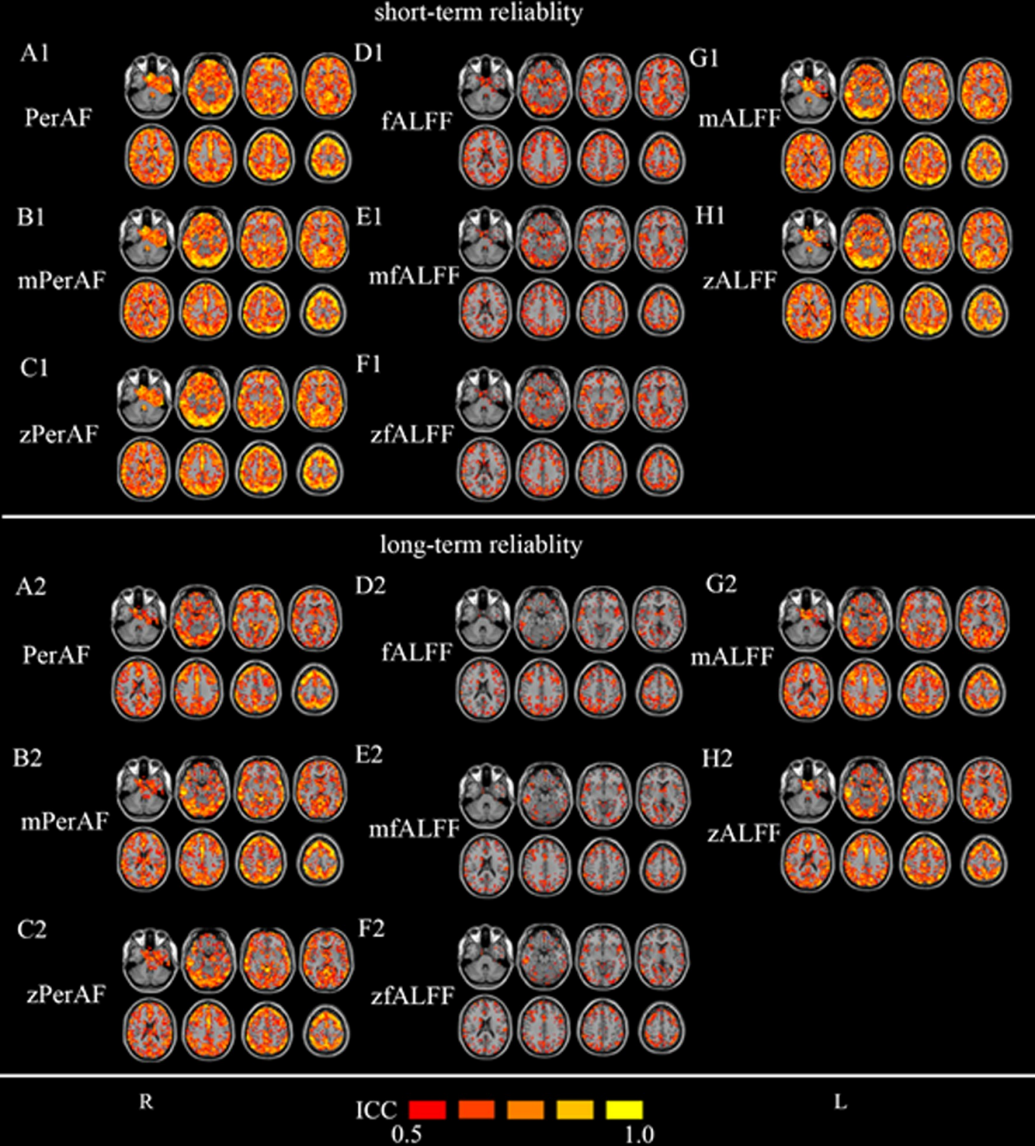
Test-retest reliability in Dataset-2. The upper part is for short-term (session 2 against session 3) (A1—H1) and lower part is for long-term (session 1 against session 2) (A2 –H2). The intraclass correlation (ICC) maps include: (A1, A2) PerAF (without standardization by global mean), (B1, B2) mPerAF (divided by the global mean PerAF), (C1, C2) zPerAF (minus mean and divided by standard deviation), (D1, D2) fALFF (without standardization by global mean), (E1, E2) mfALFF (divided by the global mean fALFF), (F1, F2) zfALFF (minus mean and divided by standard deviation), (G1, G2) mALFF (divided by the global mean ALFF), and (H1, H2) zALFF (minus mean and divided by standard deviation). The original ALFF map is mathematically unsuitable for comparison and hence not listed here. The ICC threshold was set at ≥ 0.5 for all maps. L: left side of the brain. R: right side of the brain.

To investigate the test-retest reliability of PerAF, ALFF, and fALFF, intraclass correlation coefficient (ICC) was calculated between each pair of the 3 sessions in Dataset-2. ICC has been widely used in previous studies for test-retest reliability [4,36–38]. Dataset-2 allows both long-term reliability (session 1 vs. other 2 sessions, 5–16 months apart) and short-term reliability (session 2 vs. session 3, < 1h apart). ICC > 0.5 was usually considered as moderate or higher test-retest reliability [4,36] and was also used as a threshold in this study. As shown in Fig 3, for all the metrics including PerAF, mPerAF, zPerAF, mALFF, zALFF, fALFF, mfALFF, and zfALFF, most cortical areas showed moderate to high short-term (session 2 against session 3) test-retest reliability. Long-term test-retest reliability was lower than short-term reliability (See Fig 3 for session 1 against session 2 and see (S3 Fig) for session 1 against session 3). Gray matter’s reliability was much higher than white matter. fALFF and its derivative maps showed the worst test-retest reliability among the three metrics (Fig 3).

**Fig 4:**
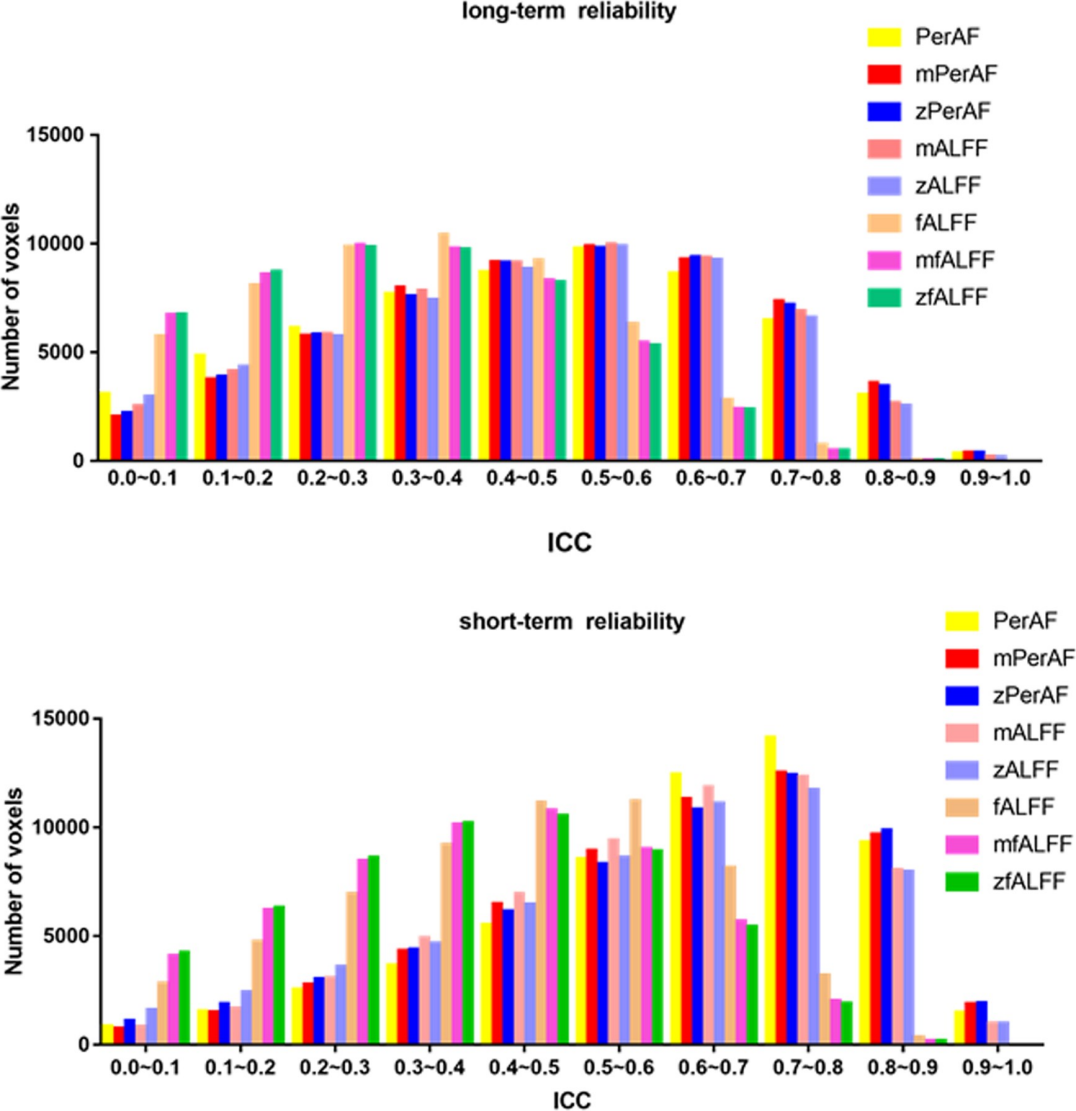
Histogram of test-retest reliability of all voxels. Y axis is the number of voxels of each bin (with an ICC step of 0.1). Upper (a) is the short-term (session 2 against session 3) reliability. In general, short-term reliability was better than long-term one. For short-term reliability, most voxels had ICC > 0.5 for all measures. Comparing the number of voxels with ICC > 0.5 among measures, PerAF, mPerAF, and zPerAF performed slightly better than mALFF and zALFF, and much better than fALFF, mfALFF, and zfALFF. Please see Table 2 for detailed number of voxels. For long-term reliability (session 1 against session 2), mPerAF and zPerAF performed similarly with (although very slightly better than) mALFF and zALFF, but PerAF (without standardization by global mean) performed worse, and fALFF, mfALFF, and zfALFF performed the worst.

We summarized the number of voxels with ICC > 0.5 of short-term (session 2 against session 3) and one long-term (session 1 against session 2) in Table 2 and Fig 4. The other long-term (session 1 against session 3) was shown in S4 Fig Generally, mPerAF and zPerAF showed similar reliability, and were better than all other metrics in both long- and short-term test-retest reliability (Fig 4). We also investigate the test-retest reliability using the data without head motion regression (Friston-24). The head motion regression (Friston-24) increased the reliability of PerAF, but decreased all other metrics (mPerAF zPerAF, mALFF, zALFF fALFF, mfALFF, mALFF, mPerAF and, zfALFF, zALFF, and zPerAF) (Table 2).

**Table 2:** Number of voxels with ICC > 0.5.

	Short-term reliability (Session 2 against session 3)		Long-term reliability (session 1 against session 2)	
	Friston-24	No-Friston-24	Friston-24	No-Friston-24
PerAF	45975	45122	28320	22793
mPerAF	44331	46336	30514	31248
zPerAF	43364	46084	30248	31743
mALFF	42629	44089	29126	30866
zALFF	40386	43510	28524	31148
fALFF	22929	26273	9961	11303
mfALFF	16907	23148	8414	12683
zfALFF	16422	22413	8244	11550

The EPI sequence is sensitive to magnetic field inhomogeneity, and hence, the mean intensity of BOLD signal may vary across scanners and scanning sessions. All ALFF, fALFF, and PerAF could be affected by the inhomogeneity. We thus calculated the variations of intensity maps across subjects and scans. It was shown that the variation across scans was much smaller than that across participants (S5 Fig). But how this variation affected the results of ALFF, fALFF, and PerAF should be further explored in future studies.

## 3. Implementation and usage of PerAF calculation toolkit

To facilitate future applications, we implemented PerAF in a new version of REST package [27] named RESTplus[28] which based on Statistical Parametric Mapping (SPM)[29]. From http://www.restfmri.net, the most recent version of RESTplus can be downloaded. The compressed package need to be extracted to a predefined directory, and then add the full path to MATLAB’s search path. Entering ‘‘restplus” in the MATLAB command window will open RESTplus’s GUI. The pipeline module can be used to calculate PerAF. It supports NIfTI image format. Users need to set the input directory. Users can put raw data or preprocessed data in input folder. The output result include PerAF (without standardization by global mean), mPerAF, and zPerAF.

We also implemented a command line in LINUX, named REST-PerAF, based on AFNI [31] for calculation of PerAF. It can be downloaded at http://www.restfmri.net.

## 4. Discussion

As a metric of spontaneous brain activity, ALFF has been widely used in RS-fMRI studies [39]. However, as shown in the simulation in the current study (Fig 1), ALFF is not a scale-independent metric. Therefore, ALFF has been standardized by the global mean value, producing mALFF or zALFF [2,4], or by the amplitude of entire frequency band (i.e., fALFF) [5]. fALFF will make the situation complex when taking sub-frequency bands into account. The current results suggest that both PerAF and mPerAF should be used in future studies.

### 4.1. Between-condition differences with and without standardization by global mean value

An EO and EC study showed that the electroencephalography (EEG) vigilance was negatively correlated with the global signal amplitude [40]. A previous study observed significant changes of normalized mean squared successive difference and variability of the successive difference between the EO–EC sessions [41]. EO and EC difference was associated with spontaneous BOLD oscillations in cortical sensory [42]. It is interesting that with and without standardization yielded quite different results. Without standardization, the areas showing significant differences in the bilateral PSMC, SMA, primary auditory cortex, and primary visual cortex became smaller (Fig 2A1). But after standardization, these clusters became smaller and two more regions, i.e., the lateral occipital cortex and orbitofrontal cortex, appeared (Fig 2B and 2C). The lateral occipital cortex plays important role in visual information processing, and the difference of fluctuation between resting EO and EC is reasonable. As PerAF, fALFF can also be used without standardization by global mean value. The global mean fALFF also showed prominent effect on the results (Fig 2D vs. 2E and 2F). These results suggest that, standardization by the global mean value has prominent effect on the results, and both results seem meaningful. Jao and colleagues found that there were some global differences of fluctuation amplitude between EO and EC [43]. We further compared the global mean PerAF between EO and EC resting states, However, there was no significant difference of the global mean PerAF between EO and EC (P = 0.8).

Taken together, although the test-retest reliability of mPerAF and zPerAF is better than PerAF, results of both with and without removing global mean PerAF seemed to be physiologically meaningful. The impact of global mean normalization could not be neglected. However, its actually physiological or psychological importance is still unclear and needs further investigation.

### 4.2. Test-retest reliability

As shown in Fig 3, all measures including PerAF, mPerAF, zPerAF, mALFF, zALFF, fALFF, mfALFF, and zfALFF showed moderate to higher short-term test-retest reliability, but the long-term reliability was not so good. These results were consistent with previous studies on the same dataset by using zALFF and zfALFF [4]. For both short- and long-term reliability (Fig 4A and 4B, Table 2), mPerAF and zPerAF showed slightly higher ICC than mALFF and zALFF, respectively. This slightly better performance may be partly contributed by normalizing the temporal mean of BOLD signal of PerAF. However PerAF had less number of voxels with ICC > 0.5 than mPerAF and zPerAF, suggesting that further standardization by global mean could improve test-retest reliability. Anyway, it does not mean that mPerAF or zPerAF could replace PerAF, as discussed in the following session of between-condition differences.

The test-retest reliability of fALFF, mfALFF, and zfALFF were much worse than PerAF, mPerAF, zPerAF, mALFF, and zALFF. These results were well consistent with the study by Zuo and colleagues [4]. The bad performance of fALFF is probably due to that it takes high frequency band into account.

### 4.3. Limitations

We proposed that PerAF is more suitable for brain lesion studies than ALFF. However, we didn’t include any data with lesions. It should be further evaluated how big effect the brain tumor or other large lesions have on global mean normalization.

## 5. Conclusions

PerAF is an analog to percent signal change widely used in task fMRI studies, and hence a straightforward metric of BOLD signal fluctuations at single voxel level. The test-retest reliability showed that mPerAF was generally slightly higher than conventional mALFF, and much better than mfALFF. The head motion regression (Friston-24) increased the reliability of PerAF, but decreased all other metrics. With and without standardization of global mean PerAF yielded quite different results, suggesting that with and without global mean standardization are not exclusive. In the future study, both PerAF and mPerAF should be used. Both results of test-retest reliability and between-condition differences suggested that PerAF is a more promising metric for RS-fMRI signal at single voxel level.

## Supporting information

S1 Fig Intersection mask of Dataset-1. The left pannel shows how many sessions (totally 34 subjects × 2 = 68 session) were covered, for each voxel in Dataset-1. The right pannel is an intersection mask which was covered by all 68 sessions.
(TIF)

S2 Fig Intersection mask of Dataset-2. The left pannel shows how many sessions (totally 25 subjects × 3 = 75 sessions) were covered, for each voxel in Dataset-2. The right pannel is an intersection mask which was covered by all 75 sessions.
(TIF)

S3 Fig The long-term test-retest reliability measured between session 1 and session 3. Only voxels with intraclass correlation (ICC) > 0.5 were shown. A: PerAF (without standardization by global mean). B: mPerAF (divided by the global mean PerAF). C: zPerAF (minus mean and divided by standard deviation). D–F: fALFF, mfALFF, and zfALFF, respectively. G, H: mALFF and zALFF, respectively. L: left side of the brain. R: right side of the brain.
(TIF)

S4 Fig The histogram of all voxels for long-term reliability of session 1 against session 3. Y axis is the number of voxels of each bin (with a step of 0.1).
(TIF)

S5 Fig Variations of intensity maps across subjects and scans. The standard deviation of normalized mean maps was calculated across subjects and scans.
(TIF)
