# Supplementary figures and images for: Percent amplitude of fluctuation: A simple measure for resting-state fMRI signal at single voxel level
Source: PLoS One. 2020 Jan 8;15(1):e0227021. doi: 10.1371/journal.pone.0227021 (PMC6948733; doi:10.1371/journal.pone.0227021)

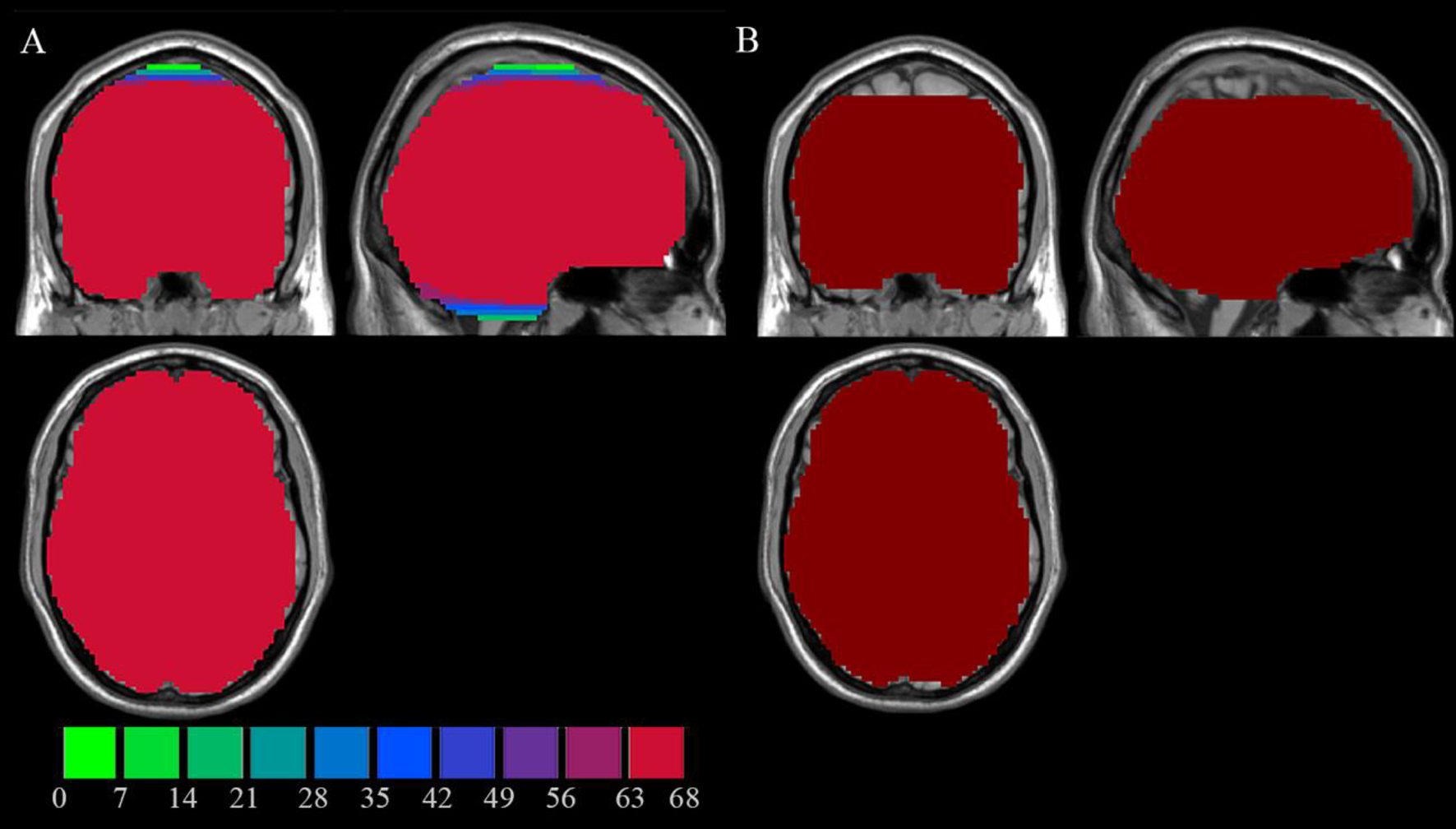

Supplement: S1 Fig — The left pannel shows how many sessions (totally 34 subjects × 2 = 68 session) were covered, for each voxel in Dataset-1. The right pannel is an intersection mask which was covered by all 68 sessions. (TIF) [file pone.0227021.s001.tif]

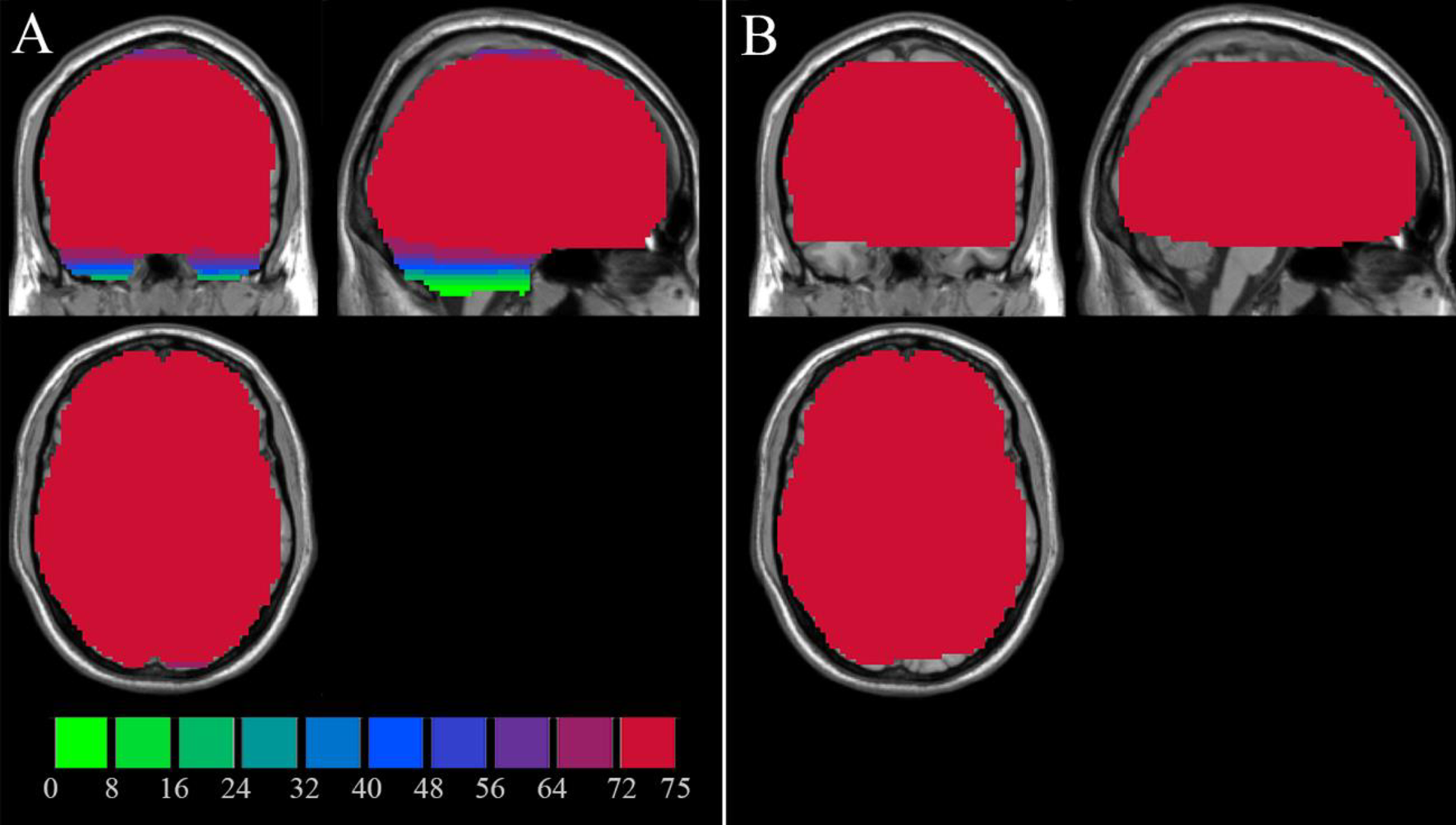

Supplement: S2 Fig — The left pannel shows how many sessions (totally 25 subjects × 3 = 75 sessions) were covered, for each voxel in Dataset-2. The right pannel is an intersection mask which was covered by all 75 sessions. (TIF) [file pone.0227021.s002.tif]

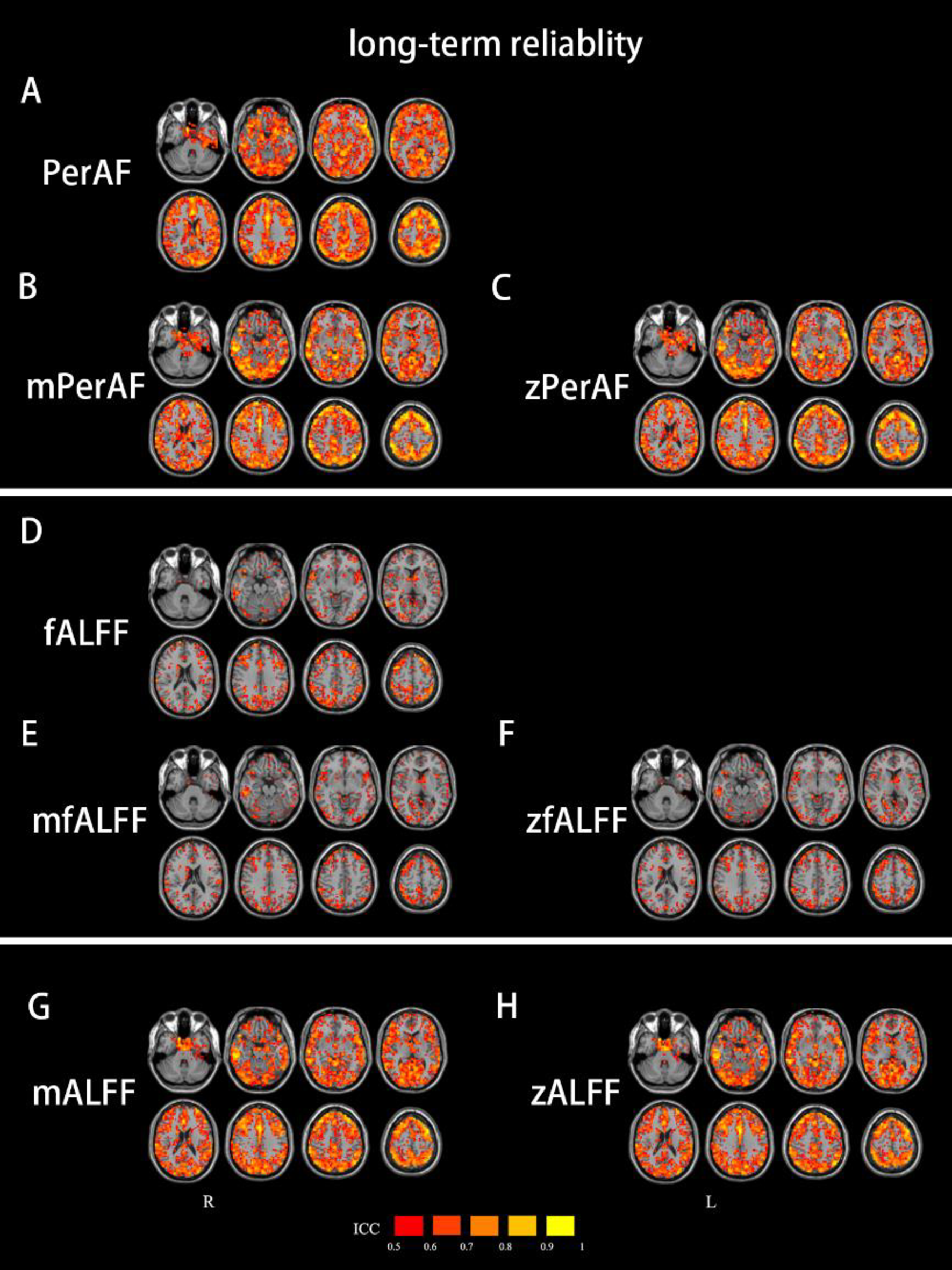

Supplement: S3 Fig — Only voxels with intraclass correlation (ICC) > 0.5 were shown. A: PerAF (without standardization by global mean). B: mPerAF (divided by the global mean PerAF). C: zPerAF (minus mean and divided by standard deviation). D–F: fALFF, mfALFF, and zfALFF, respectively. G, H: mALFF and zALFF, respectively. L: left side of the brain. R: right side of the brain. (TIF) [file pone.0227021.s003.tif]

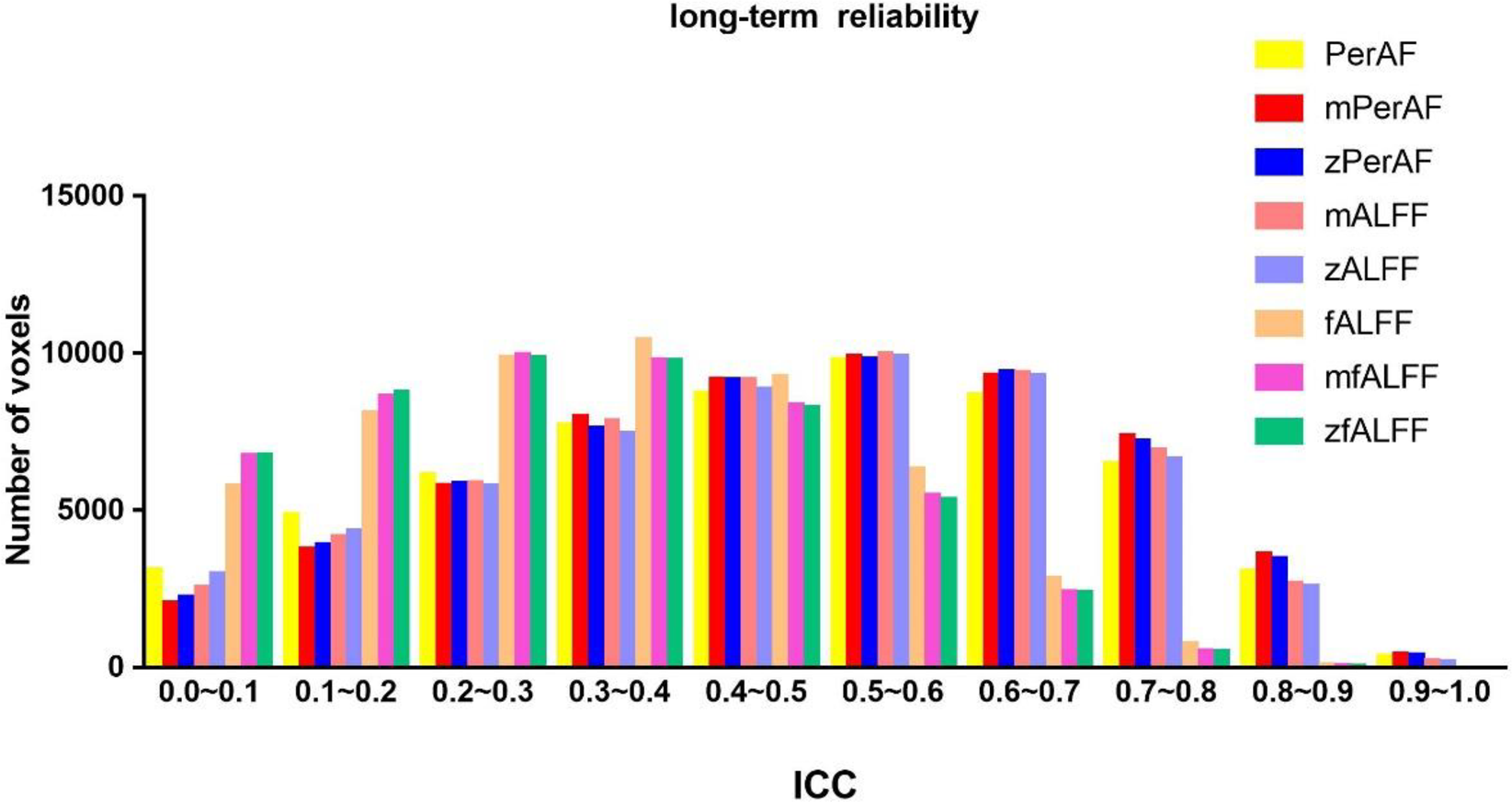

Supplement: S4 Fig — Y axis is the number of voxels of each bin (with a step of 0.1). (TIF) [file pone.0227021.s004.tif]

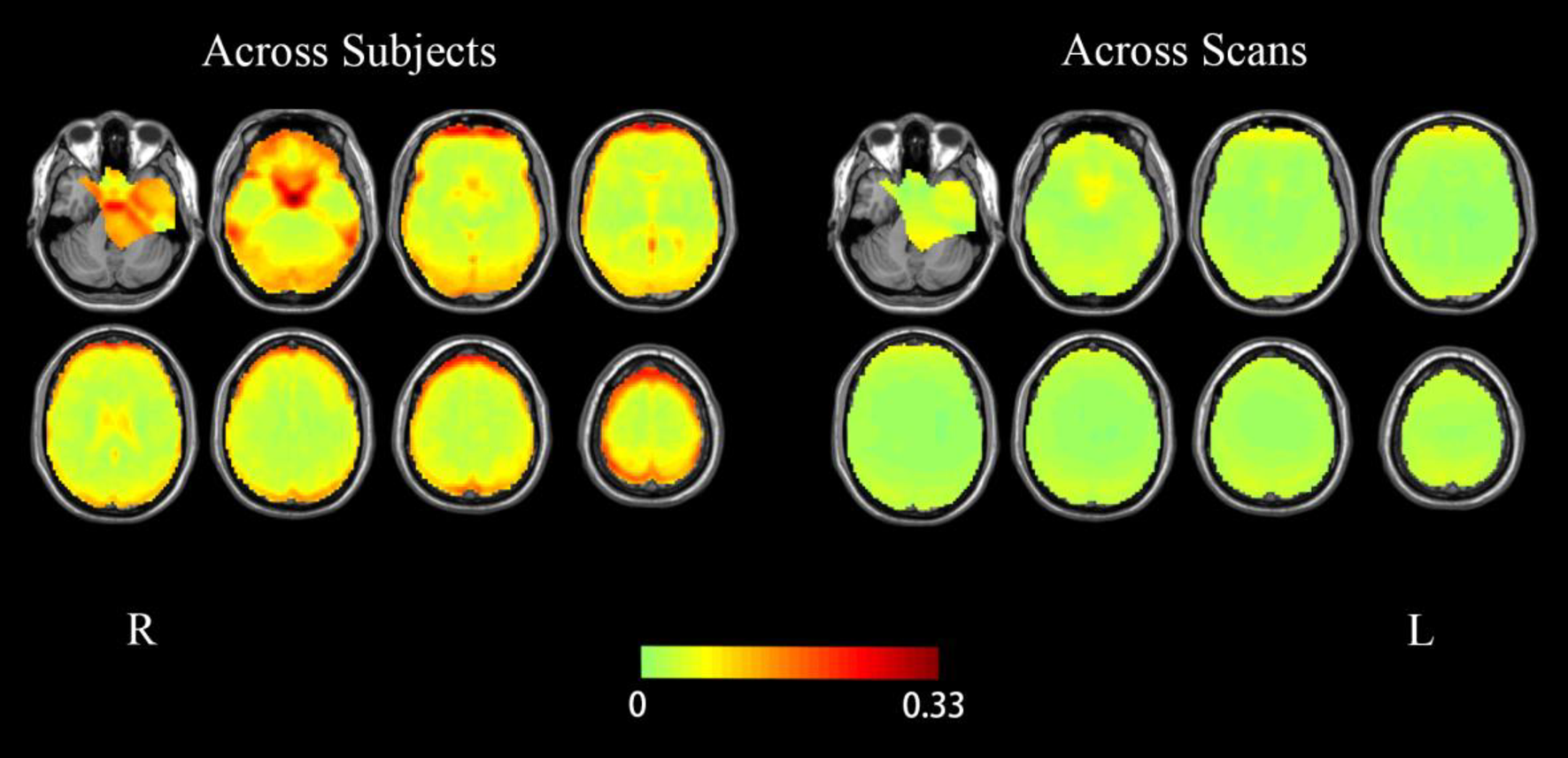

Supplement: S5 Fig — The standard deviation of normalized mean maps was calculated across subjects and scans. (TIF) [file pone.0227021.s005.tif]
